# A multifaceted approach to understanding bat community response to disturbance in a seasonally dry tropical forest

**DOI:** 10.1038/s41598-021-85066-z

**Published:** 2021-03-11

**Authors:** Darwin Valle, Daniel M. Griffith, Andrea Jara-Guerrero, Diego Armijos-Ojeda, Carlos I. Espinosa

**Affiliations:** 1 Maestría de Biología de la Conservación y Ecología Tropical, Departamento de Ciencias Biológicas, Universidad Técnica Particular de Loja, 1101608 Loja, Ecuador; 2 EcoSs_Lab, Departamento de Ciencias Biológicas, Universidad Técnica Particular de Loja, 1101608 Loja, Ecuador

**Keywords:** Ecology, Biodiversity, Tropical ecology

## Abstract

Given widespread habitat degradation and loss, reliable indicators are needed that provide a comprehensive assessment of community response to anthropogenic disturbance. The family Phyllostomidae (Order Chiroptera) has frequently been the focus of research evaluating bats’ response to habitat disturbance in seasonally dry tropical forests (SDTFs). However, few studies compare this family to the larger bat assemblage to assess its efficacy as a bioindicator. We compared community and species-specific attributes of understory phyllostomid and all understory bat species: (1) along a gradient of habitat disturbance within a human-modified SDTF landscape; and (2) between forest and riparian habitats within each disturbance level. We captured 290 individuals belonging to 13 species and 4 families. Phyllostomid species exhibited greater sensitivity to disturbance than the understory bat community as a whole based on richness and beta diversity. Both groups were more sensitive to disturbance in forest than riparian habitat, but phyllostomid species were more likely to be lost from highly disturbed forest habitat. The two dominant species declined in abundance with disturbance but variation in body condition was species-specific. These results suggest that Phyllostomidae are more effective indicators of human disturbance in SDTF than the understory bat community as a whole and evaluation of bats’ response to disturbance is best accomplished with a multifaceted approach.

## Introduction

Ecosystem structure and functionality are changing worldwide due to the increasing intensity and extent of human activities^1^. A main driver of these changes is the conversion of natural habitat to commodity-based agriculture, pasture, shifting agriculture, and tree plantations^2^. However, understanding the impacts of habitat conversion and loss on natural communities is complex given that species—even those that are closely related—respond differently to similar anthropogenic disturbances, which can blur response signals at the community level^3–6^. We need reliable methods and indicators that enable the detection, measurement, and prediction of the effects of anthropogenic disturbance on biodiversity and provide a comprehensive assessment of community response^7^.

The Order Chiroptera has been proposed as an effective indicator of ecosystem integrity given that bats play important roles as ecosystem service providers and respond quickly to anthropogenic disturbances^4,8,9^. However, bats have exhibited contrasting responses to disturbances, calling into question their reliability as bioindicators as a group^7,10,11^. This has led researchers to use and test taxonomic subgroups and functional guilds within Chiroptera as indicators of habitat disturbance^12–14^. The family Phyllostomidae has been the focus of many studies evaluating bats’ response to habitat disturbance given this group’s specialized feeding requirements and high taxonomic and functional diversity^10,15–17^. Phyllostomid species comprise the most diverse Neotropical bat family and exhibit a wide range of responses to disturbance depending on the attribute measured, disturbance type, and region^18^. However, since this family was first proposed as a promising indicator of habitat disturbance^17^, few studies have directly compared attributes of an entire bat assemblage or at least a larger portion of the bat community with those of Phyllostomidae to assess its efficacy as a bioindicator. In a meta-analysis of 149 bat species based on 1115 study cases from the Neotropics, phyllostomid species were positively associated with human-impacted areas compared to intact forests, except in the case of silvopastoral systems and intensive monocultures^12^. Yet while this study compared species’ occurrences between different land uses, it did not take into account other species-specific or assemblage-based attributes nor did it directly compare the family to the larger bat community under a specific set of environmental conditions. Such comparisons are necessary to determine the degree to which phyllostomid bats are a reliable bioindicator in different environmental and disturbance contexts.

As in other ecosystems, phyllostomid species have been the focus of bat research in seasonally dry tropical forests (SDTFs), where their response to disturbance has been compared across different habitats, spatial scales, and levels of biological complexity ranging from individuals to the community^10,18–22^. While evidence suggests that Phyllostomidae as a family is a poor bioindicator given the contrasting responses of its members to disturbance^7^, the question remains for STDFs where bat research is limited compared to other ecosystems^19^. In a study of SDTF successional stages in Mexico, Venezuela and Brazil, Avila-Cabadilla et al.^20^ attributed differential disturbance responses of phyllostomid bats to regional differences in environmental conditions, bat species composition, and landscape characteristics. However, other studies show more consistent responses such as decreasing phyllostomid richness from late to early successional habitats^10,19,21^. In general, the question of whether Phyllostomidae is a reliable indicator of disturbance has not been adequately addressed in SDTFs.

Depending on the intensity and duration, a disturbance does not necessarily provoke changes in richness but can induce species turnover, shifts in relative abundances, or simply changes in individual fitness^23^. For example, Estrada and Coates-Estrada^24^ and Zarazúa-Carbajal et al.^22^ found changes in the relative dominance of bat species but not richness between different levels of habitat disturbance in a Mexican rainforest and SDTF, respectively. Shifts in bat communities resulting from disturbance can largely be attributed to species-specific characteristics and habitat affinities^7,25^. Within the same community, species with different habitat requirements and dispersal ability can exhibit different levels of sensitivity to disturbance^11,15^. The most sensitive species are at greatest risk of local extinction, while generalist species may actually increase in population size^5,26^. Between these two extremes, moderately sensitive species may suffer deterioration in individual health, which can lead to low reproductive capacity and population decline^27^. To understand a community’s response to disturbance comprehensively, measures such as assemblage composition, relative abundance, and individual condition should be assessed in addition to species richness.

Studies of disturbance gradients demonstrate that habitats characterized by high vegetation diversity and structural complexity such as gallery forests, agroforestry systems, and live fences are especially important for bats in human-modified landscapes^14,28,29^. Riparian vegetation, which is usually maintained in such landscapes where agriculture is non-intensive, is crucial for many bats as a source of resources as well as flyways, which enhance landscape connectivity^24,28,30,31^. In highly seasonal environments such as SDTFs, habitat degradation can trigger a significant reduction in bat diversity due to heat and water stress^10^. By providing resources and shelter, riparian habitats provide a key refuge for bats in the midst of adverse climatic conditions^28^ and can actually increase the abundance of disturbance-sensitive species in SDTF, especially frugivores^18,22^. Studies evaluating bats’ response to disturbance should thus take into account habitat elements such as riparian vegetation that can buffer the most severe effects of habitat degradation^18^.

To assess the effectiveness of Phyllostomidae as a bioindicator and more fully understand the response of bats to human disturbance in SDTF, we used a multi-pronged approach that compared assemblage-level and species-specific responses of understory phyllostomid bats and the understory community as a whole to habitat disturbance. We tested three hypotheses to formulate a comprehensive assessment of bats’ response to disturbance in a human-modified landscape. First, given the sensitivity of many phyllostomids to reduced structural complexity and loss of zoochorous plants associated with habitat degradation in SDTF^10,32^, we expected phyllostomid richness to decline more precipitously than that of the understory bat community as a whole with habitat disturbance. For the same reason, we expected species loss and abundance declines to contribute more to compositional differences between disturbance levels than species turnover and abundance variation for Phyllostomidae compared to the entire bat community. Second, as species-specific responses to disturbance can manifest as changes in abundance and individual health, we expected abundance and body condition to decrease with disturbance and that the magnitude of this decline would vary by species. Third, because riparian habitat can serve as a refuge for bats in degraded SDTF, we expected changes in community attributes along the disturbance gradient to have a weaker signal in riparian habitat relative to forest habitat. We compared several attributes of both Phyllostomidae and the overall understory bat community to determine the best indicators of ecosystem integrity in SDTF.

## Results

During 24,786 mist-net-meter-hours, we captured a total of 290 individuals belonging to 13 species, seven foraging guilds, and four families: Molossidae, Noctilionidae, Phyllostomidae and Vespertilionidae (Table 1). Phyllostomidae was the most speciose and abundant family with eight species (61.5% of all species) and 271 individuals (93.5% of all captures). The two most abundant species were *Artibeus fraterculus* (66.9% of all individuals) and *Desmodus rotundus* (18.3% of all individuals), which are both phyllostomids, while the remaining species were each represented by ten or fewer individuals. Analysis of a guild-specific response at the ensemble level was limited to aerial and gleaning insectivores given that all other guilds were represented by only one or two species. Despite the greater abundance of aerial insectivores relative to gleaning insectivores in semi-natural and degraded forests, the frequencies of these two guilds were independent of habitat disturbance (Fisher´s exact test, *p* = 0.086). No individual was recaptured during the same sampling session at a given site.

**Table 1:** Total number of bats captured in three levels of habitat disturbance (numbers outside parentheses) and in forest and riparian habitat nested within each disturbance level (numbers within parentheses corresponding to forest/riparian).

FAMILY Subfamily *Species* (Guild)	Level of habitat disturbance			Total
	Natural forest	Semi-natural forest	Degraded forest	
**MOLOSSIDAE**				
** Molossinae**				
* Molossus molossus* (AI)	1 (0/1)	2 (0/2)	2 (1/1)	5
**NOCTILIONIDAE**				
* Noctilio leporinus* (C)	0	1 (0/1)	2 (0/2)	3
**PHYLLOSTOMIDAE**				
** Desmodontinae**				
* Desmodus rotundus* (S)	28 (6/22)	19 (3/16)	6 (2/4)	53
** Glossophaginae**				
* Glossophaga soricina* (N)	3 (1/2)	2 (2/0)	5 (1/4)	10
** Phyllostominae**				
* Chrotopterus auritus* (C)	0	2 (0/2)	0	2
* Gardnerycteris crenulatum* (GI)	3 (1/2)	0	0	3
* Lophostoma occidentalis* (GI)	3 (0/3)	2 (1/1)	0	5
* Micronycteris megalotis* (GI)	3 (2/1)	0	0	3
* Phyllostomus discolor* (O)	1 (1/0)	0	0	1
** Stenodermantinae**				
* Artibeus fraterculus* (F)	91 (47/44)	65 (42/23)	38 (17/21)	194
**VESPERTILIONIDAE**				
** Myotinae**				
* Myotis nigricans* (AI)	2 (1/1)	2 (0/2)	2 (0/2)	6
* Myotis riparius* (AI)	1 (0/1)	0	1 (0/1)	2
** Vespertilioninae**				
* Rhogeessa velilla* (AI)	3 (3/0)	0	0	3
Total abundance	139 (62/77)	95 (48/47)	56 (21/35)	290
Richness	11 (8/9)	8 (4/7)	7 (4/7)	13
Number of sites	8	5	8	21
Sampling effort (mist-net-meter-hours)	8262	8262	8262	24,786

Species guild assignations are based on Brito et al.^33^ and Tirira^34^: aerial insectivore (AI), carnivore (C), frugivore (F), gleaning insectivore (GI), nectarivore (N), omnivore (O) and sanguivore (S).

**Figure 1:**
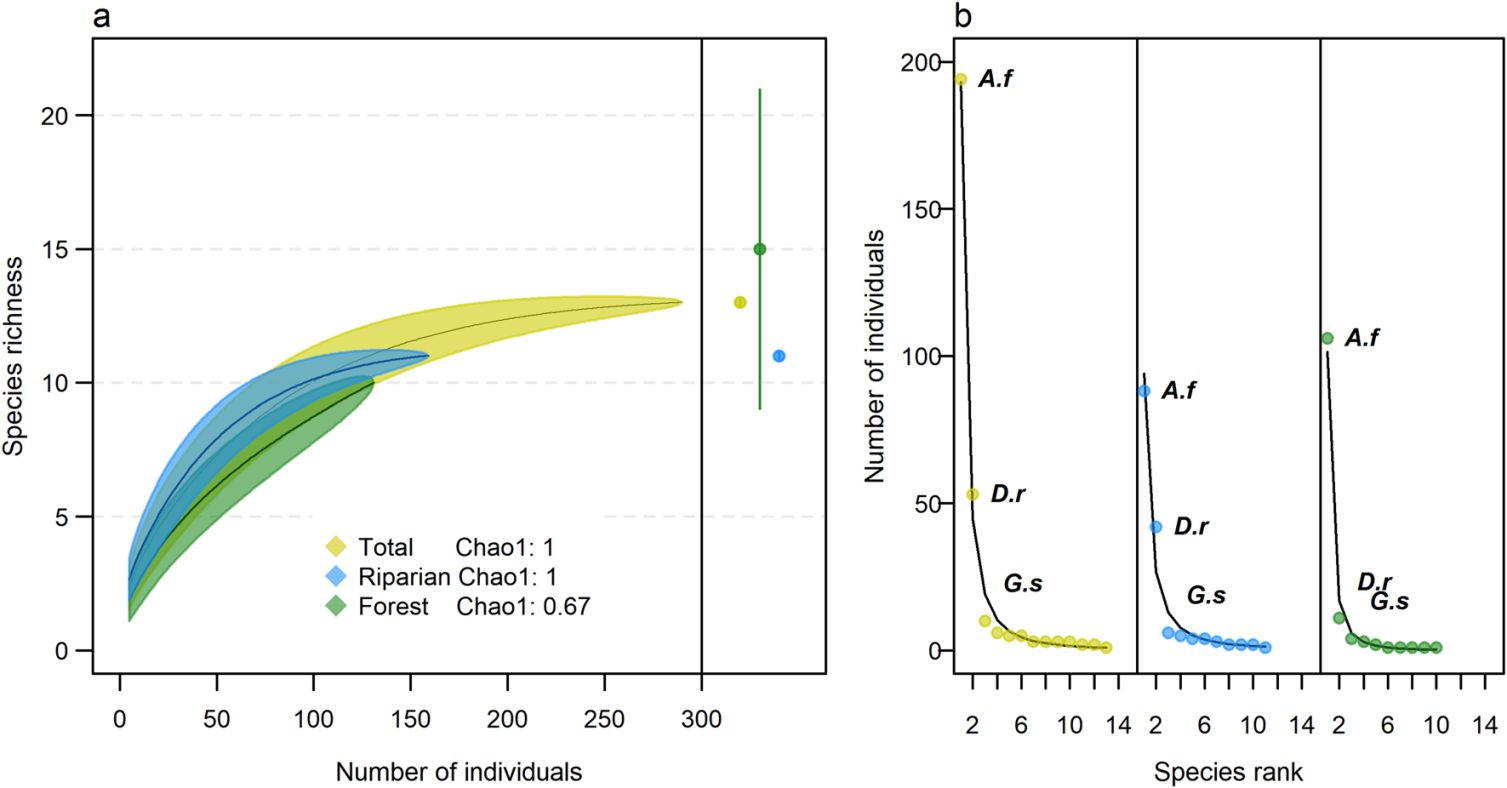
Understory bat community structure for the entire study area and forest and riparian habitats nested within sites showing: (**a**) species accumulation curves with 95% confidence intervals based on individual-based rarefaction, Chao1 richness estimates of the complete assemblage in each habitat with 95% confidence intervals (points on right), and the completeness index calculated from the Chao1 estimator; and (**b**) species’ rank abundances. Initials correspond to species names: *Artibeus fraterculus (A.f.), Desmodus rotundus (D.r.),* and *Glossophaga soricina (G.s.).* Colors in (**b**) correspond to the entire study area, riparian habitat, and forest habitat as in (**a**).

We registered eleven species in riparian habitat and ten species in forest habitat (Table 1). Neither rarefaction nor the Chao1 estimate showed significant differences in overall species richness between these habitats based on overlapping confidence intervals (Fig. 1a). The completeness index showed complete sampling efficiency in riparian habitat but low efficiency in forest habitat, implying that further sampling could result in higher richness in the latter.

Rank abundance patterns were similar in forest and riparian habitats as *A. fraterculus*, *D. rotundus* and *Glossophaga soricina* were the first, second and third ranking species in both habitats, respectively (Fig. 1b). Three species were found exclusively in riparian habitat (*Chrotopterus auritus*, *Myotis riparius*, *Noctilio leporinus*) and two in forest habitat (*Phyllostomus discolor*, *Rhogeessa velilla*).

### Richness of all species and phyllostomid species between disturbance levels

**Figure 2:**
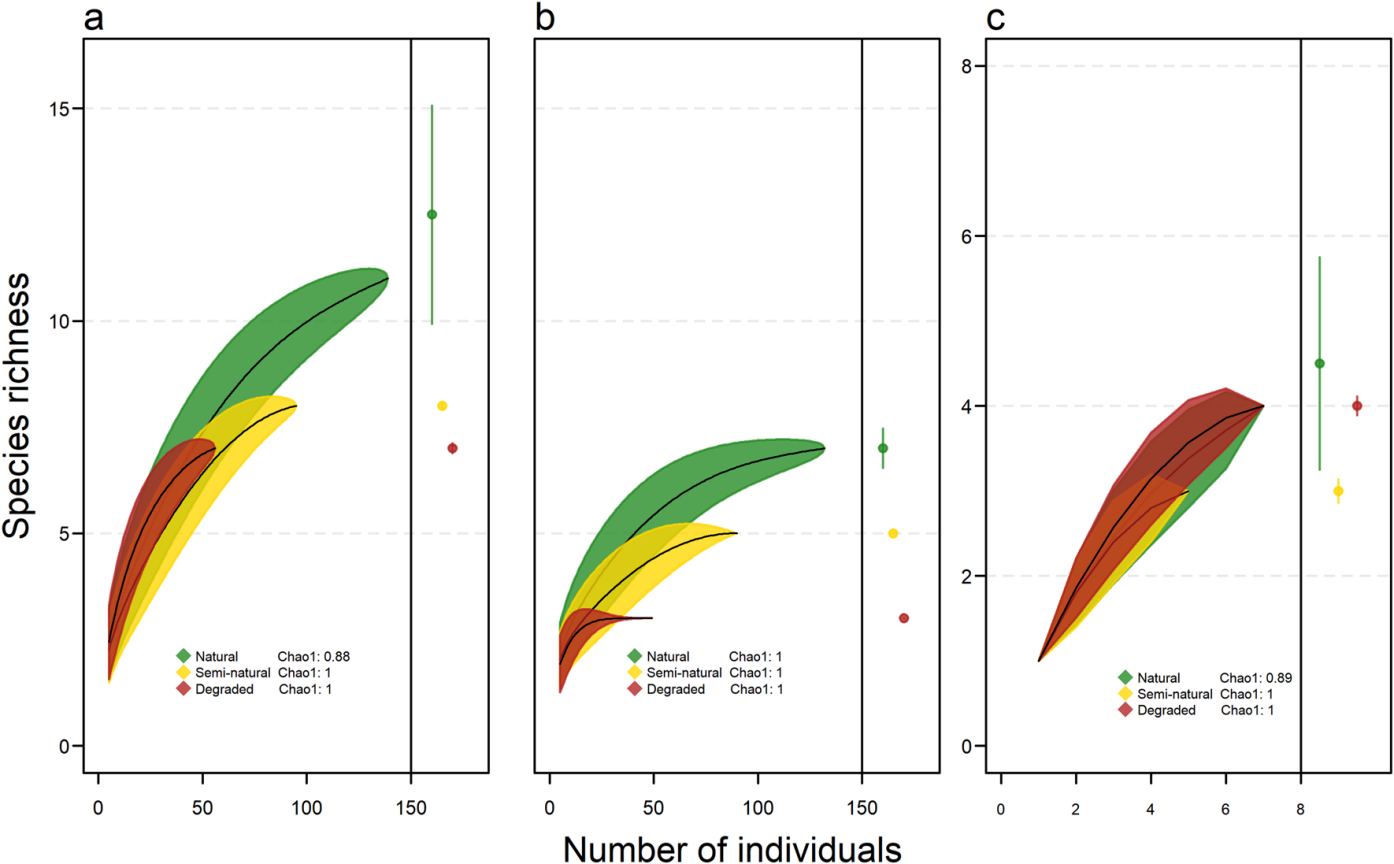
Bat richness with 95% confidence intervals based on individual-based rarefaction and Chao1 richness estimates between three levels of habitat disturbance for: (**a**) all understory species, (**b**) understory phyllostomid species, and (**c**) understory non-phyllostomid species. The completeness index calculated from the Chao1 estimator for each disturbance level is shown.

Overall species richness declined along the gradient of habitat disturbance from 11 species in natural forest, eight species in semi-natural forest, to seven species in degraded forest (Table 1; Fig. 2a). Species richness was significantly higher in natural than semi-natural forest based on non-overlapping 95% confidence intervals of the rarefaction curves, but did not differ significantly between natural and degraded forest or semi-natural and degraded forest. However, species richness is not expected to increase substantially with further sampling in degraded or semi-natural forest given that completeness was equal to one for these disturbance levels. Chao1 estimates indicated that richness of the whole understory assemblage was different between all three disturbance levels (albeit marginally so between semi-natural and degraded forest).

The decline in richness along the disturbance gradient was more pronounced and clearly different between all three disturbance levels for phyllostomid species based on non-overlapping confidence intervals and sampling efficiency equal to one for all levels (Fig. 2b). Phyllostomid richness declined from seven species in natural forest, five species in semi-natural forest to three species in degraded forest. In contrast, richness of non-phyllostomid species did not differ significantly between disturbance levels given the high overlap among confidence intervals, although the curves were based on very few individuals and should be interpreted with caution (Fig. 2c).

### Richness between disturbance levels in forest and riparian habitats

**Figure 3:**
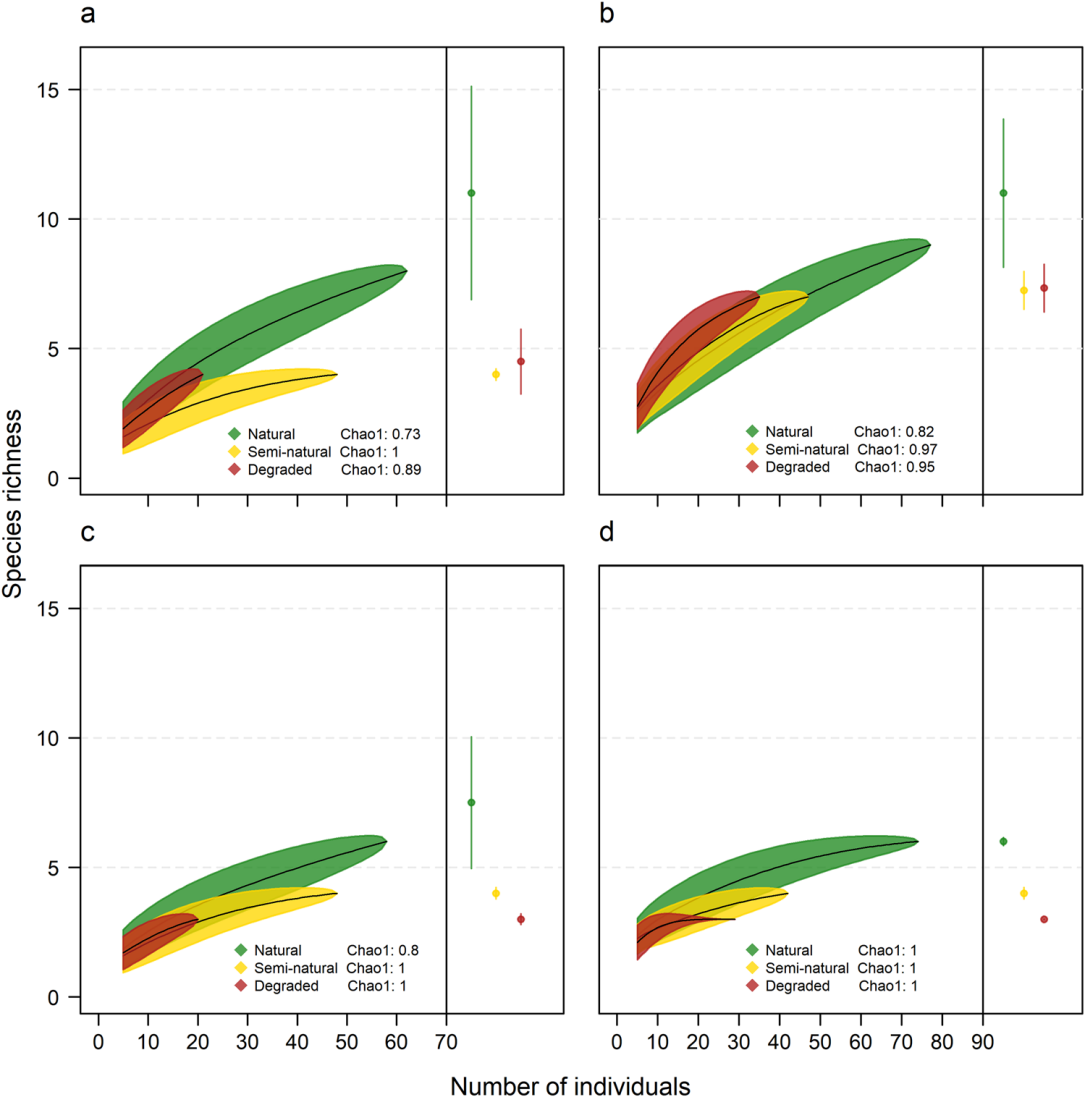
Bat richness with 95% confidence intervals based on individual-based rarefaction and estimated richness based on Chao1 between three levels of habitat disturbance for: (**a**) all understory species in forest habitat, (**b**) all understory species in riparian habitat, (**c**) understory phyllostomid species in forest habitat, and (**d**) understory phyllostomid species in riparian habitat. The completeness index calculated from the Chao1 estimator for each disturbance level is shown.

Rarefied richness of all understory species was significantly higher in natural than semi-natural forest when assemblages were compared in forest habitat (Fig. 3a), but was not different between disturbance levels in riparian habitat (Fig. 3b). Estimated richness was significantly higher in natural than semi-natural and degraded forest in forest habitat and higher in natural than semi-natural forest in riparian habitat, while semi-natural and degraded forests were not different from each other in either habitat. In contrast, rarefied richness of phyllostomid species was significantly higher in natural than semi-natural forest and estimated richness was significantly different between all three disturbance levels in both forest and riparian habitats (Fig. 3c,d).

### Beta diversity between disturbance levels

**Figure 4:**
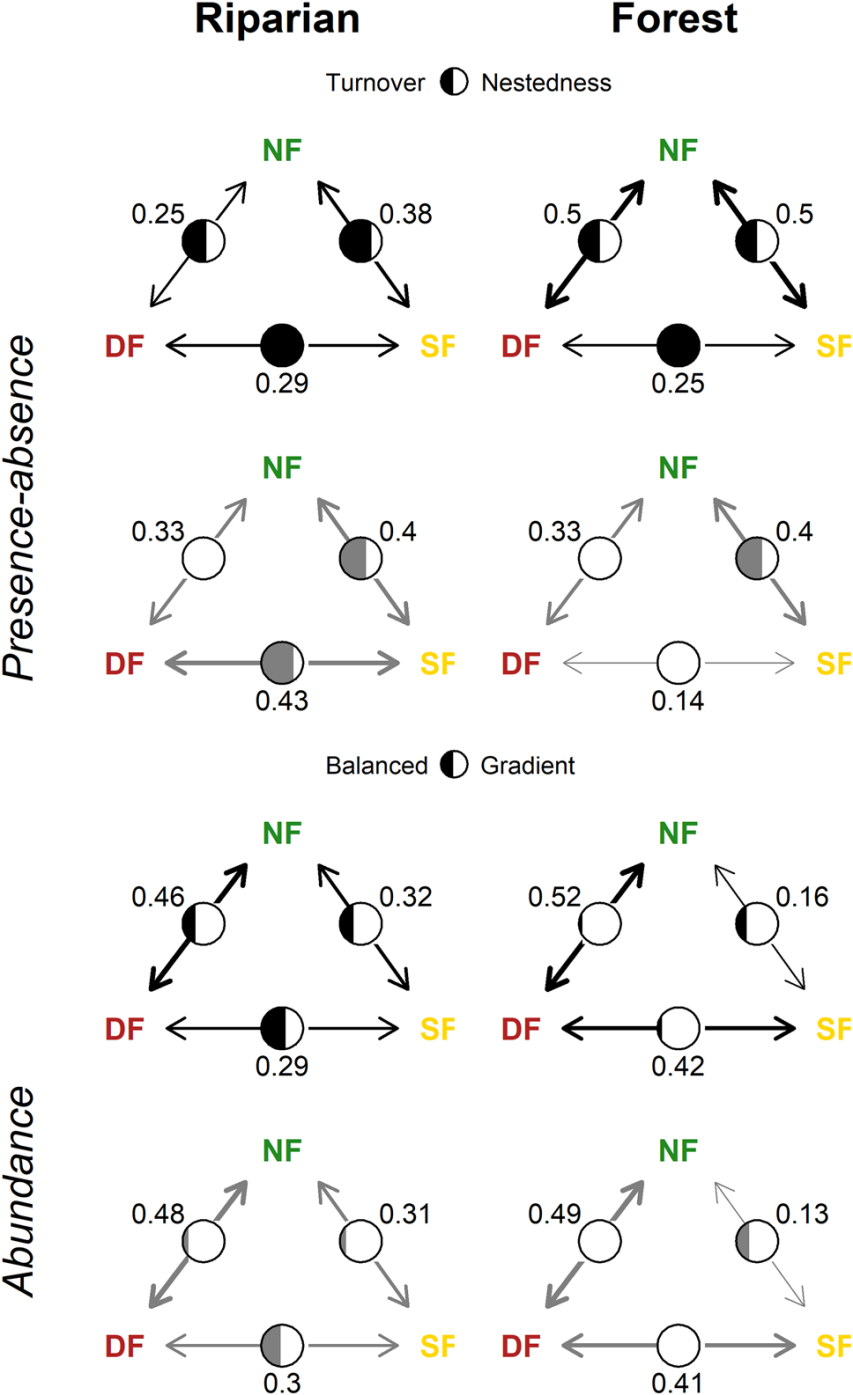
Beta diversity of understory bats between natural forest (NF), semi-natural forest (SF), and degraded forest (DF) in riparian and forest habitat based on presence-absence and abundance data for all species (black circles) and phyllostomid species (gray circles). Incidence-based beta diversity was partitioned into species turnover and nestedness^46^. Abundance-based beta diversity was partitioned into balanced variation in species abundances and abundance gradients^47^.

For both all understory species and phyllostomid species, rankings of beta diversity among disturbance levels differed between forest and riparian habitat depending on whether incidence or abundance data were examined. Based on incidence data, beta diversity (ß_sor_) of all species was highest between natural and semi-natural forest in riparian habitat (Fig. 4). In this habitat type, species turnover (ß_sim_) contributed more to beta diversity than nestedness (ß_nes_) in the natural versus semi-natural and semi-natural versus degraded forest comparisons, whereas nestedness was relatively more important between natural and degraded forest. In forest habitat, by contrast, beta diversity of all species was generally higher than that in riparian habitat and the same (0.50) between natural versus semi-natural and natural versus degraded forests, with equal contributions from turnover and nestedness. Beta diversity was lowest between semi-natural and degraded forest, which was due exclusively to species turnover.

For phyllostomid species, patterns of incidence-based beta diversity were different from those of the entire understory community due to the greater importance of nestedness. Similar to all species, phyllostomid beta diversity was high and due mainly to species turnover between natural and semi-natural forest in both riparian and forest habitats (Fig. 4). Unlike the whole community, however, nestedness was the exclusive factor underlying phyllostomid beta diversity between natural and degraded forest in both habitats and between semi-natural and degraded forest in forest habitat.

Contrary to dissimilarities based on presence-absence, beta diversity based on abundance (ß_Baselga B-C_) was nearly identical for the whole understory community and phyllostomid species, with the highest scores between natural and degraded forest in both riparian and forest habitats (Fig. 4). Beta diversity was lowest between natural versus semi-natural forest in forest habitat, but relatively similar between natural versus semi-natural and semi-natural versus degraded forest in riparian habitat. For both all species and phyllostomid species, abundance gradients contributed substantially more to beta diversity than balanced variation in abundance, with the exception of semi-natural versus degraded forest in riparian habitat.

### Abundance and body condition of the two dominant species

**Figure 5:**
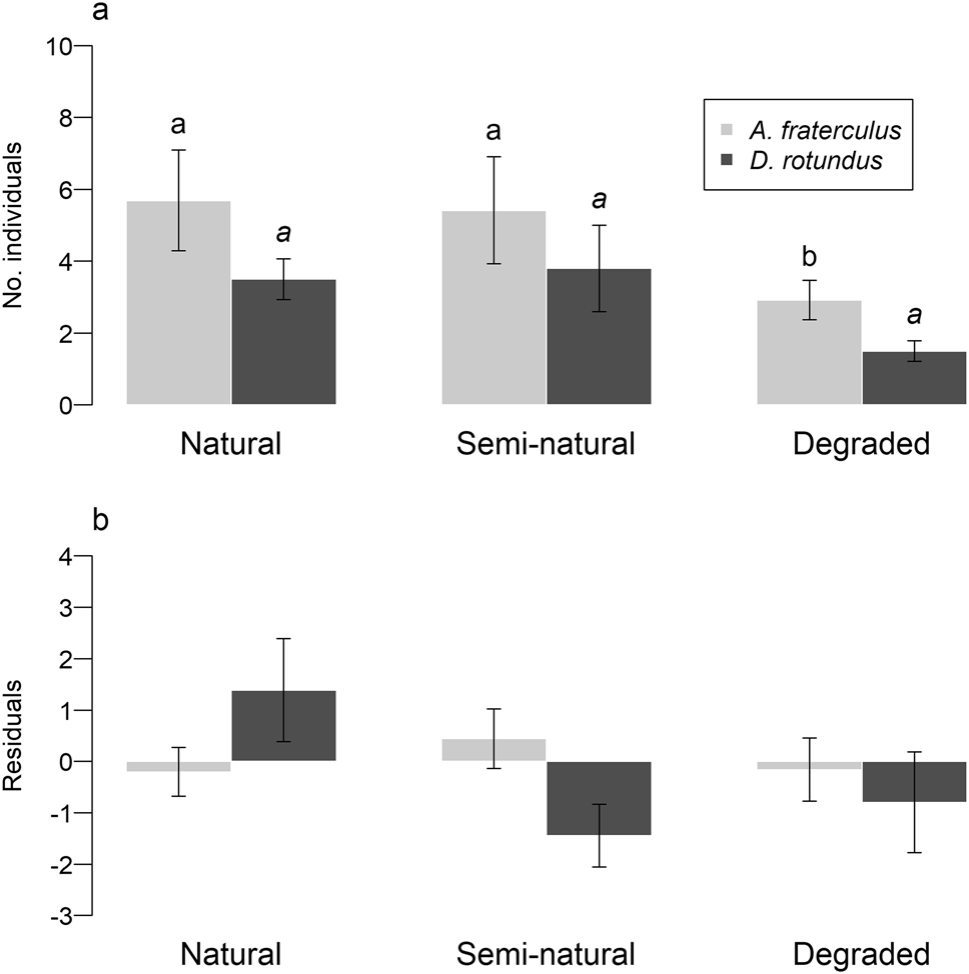
Response of *A. fraterculus* and *D. rotundus* to habitat disturbance in terms of (**a**) abundance and (**b**) body condition. Body condition was measured as the standardized residuals of a linear regression model analyzing body mass as a function of forearm length. Different letters indicate significant differences between levels of habitat disturbance. Standard error bars are shown.

*A. fraterculus* was significantly less abundant in degraded than natural and semi-natural forest, which were not significantly different from one another (Fig. 5a). *D. rotundus* did not differ significantly in abundance between disturbance levels, although it was also least numerous in degraded forest. Body condition did not vary significantly with disturbance level for either species (Fig. 5b). However, each species exhibited a different pattern along the disturbance gradient. Body condition of *A. fraterculus* was highest in semi-natural forest, whereas that of *D. rotundus* was highest in natural forest and lowest in semi-natural forest.

## Discussion

Phyllostomid species exhibited greater sensitivity to disturbance in SDTF than the understory bat community as a whole based on both richness and beta diversity. While both groups were more sensitive to disturbance in forest than riparian habitat, phyllostomid species were more likely to be lost from highly disturbed (i.e., degraded) forest habitat, providing a clearer response signal and reinforcing the importance of riparian habitat for bats in human-modified SDTF landscapes. These results support the argument that phyllostomid species are more effective indicators of anthropogenic disturbance in SDTF than the understory bat community as a whole and that robust evaluation of bat community response to disturbance is best accomplished with a multifaceted approach.

In support of the first hypothesis, Phyllostomidae showed greater sensitivity to habitat disturbance compared to the understory community as a whole based on richness. The negative association between richness and disturbance for both groups was likely the result of reduced tree density in semi-natural and degraded forests compared to natural forest, caused by years of selective logging, agriculture, and livestock grazing^8,10,19,24,35,36^. Simplification of structural complexity can adversely affect the presence and abundance of bats by reducing their roosting sites, shelter and food availability^31,37^. Yet while richness of both the whole understory community and understory phyllostomids decreased significantly with habitat disturbance, this decline was more pronounced for phyllostomids. Five of the eight phyllostomid species detected in the study were absent from degraded forest (Table 1). Notably, all five of these species belong to the subfamily Phyllostominae and were all rare, each comprising < 2% of total captures. This rareness, along with the intolerance of many Phyllostominae to habitat modification^8,23^, makes them especially vulnerable to local extinction due to stochastic events, which can be exacerbated by habitat deterioration^38,39^. These species’ sensitivity to disturbance is consistent with that found in previous studies for *Micronycteris megalotis*^17^, *C. auritus*^24,40^, and *Gardnerycteris crenulatum*^15^*.* On the other hand, four of the five non-phyllostomid species captured in the study—and seven of the eight non-Phyllostominae species—were found in semi-natural and/or degraded forest, implying that these species were more tolerant of disturbance than Phyllostomidae. The fact that the four non-phyllostomid species showing tolerance to disturbance were captured primarily in riparian habitat (Table 1) suggests that their prey—aerial insects in the case of *Molossus molossus*, *M. nigricans* and *M. riparius* and fish in the case of *N. leporinus*—were little affected by habitat disturbance as long as water was available^41^. Thus phyllostomid richness, due mainly to the sensitivity of the subfamily Phyllostominae to disturbance, provided a better indicator of ecosystem integrity than richness of non-phyllostomid species and of the understory bat community as a whole.

Although the number of species captured was small and our sample was dominated by two species, which is typical of bat assemblages in SDTFs^10,19,22,42^, the use of individual-based rarefaction and richness estimation to account for undersampling bias as well as the high sampling efficiency indicated that the differences in richness detected between disturbance levels were fairly robust^43,44^. Even when disturbance levels were separated into forest and riparian habitats, the completeness index calculated from the Chao1 estimator was high except for the natural forest level (Fig. 3a–c). Yet this suggests that the pattern of higher richness in natural forest can also be considered robust as further sampling would likely result in the detection of more species at this level compared to the semi-natural and degraded levels. Given this differential sampling efficiency between disturbance levels and the fact that Chao1 provides the *minimum* number of species in an assemblage^43,45^, we recommend further sampling to corroborate these results and determine the degree to which richness is highest in natural forest.

Divergent beta diversity patterns also provided evidence that phyllostomid species are more sensitive to habitat disturbance than the understory community as a whole. While richness can mask compositional shifts between assemblages, the partitioning of ß_sor_ into species turnover and nestedness shed light on the processes underlying biodiversity patterns, namely species replacement and species loss^46^. In terms of species presence-absence, the most important compositional shift occurred between natural and semi-natural forest. Beta diversity of all understory species and phyllostomids was high and driven primarily by species replacement between these two disturbance levels in both riparian and forest habitats. In contrast, the dominant process underlying beta diversity between semi-natural and degraded forest differed by taxonomic group and habitat. For the entire understory community, species replacement was the exclusive process in both habitats. In riparian habitat, two species in semi-natural forest (*C. auritus* and *Lophostoma occidentalis*) were replaced by two species in degraded forest (*G. soricina* and *M*. *riparius*), while in forest habitat one species in semi-natural forest (*L. occidentalis*) was replaced by another in degraded forest (*M. molossus*). For phyllostomid species, however, species replacement was more important in riparian habitat (*C. auritus* and *L. occidentalis* were replaced by *G. soricina*), while species loss was the exclusive driver of beta diversity in forest habitat (*L. occidentalis* dropped out).

Likewise, when natural and degraded forests were compared, compositional changes were driven nearly equally by replacement and loss for the whole understory community, whereas loss was the exclusive process driving understory phyllostomid dissimilarity. The fact that no species was found exclusively in degraded forest suggests that this disturbance level acted as a filter for disturbance-intolerant species, particularly the subfamily Phyllostominae, and failed to contribute any new species distinct from those found in natural or semi-natural forests. Given the general affinity of Phyllostomidae for human-impacted habitats except intensive monocultures and pastures^12^, this result suggests that degraded sites were disturbed to such a degree as to exclude this family except for its most abundant members (i.e., *A*. *fraterculus*, *D*. *rotundus* and *G*. *soricina*). As with richness, the greater role of species loss as opposed to replacement in inducing compositional shifts implied that phyllostomids were more sensitive to habitat disturbance than the understory bat community as a whole. Furthermore, the disturbance response based on foraging guild could not be disentangled from taxonomic group. All three gleaning insectivores were sensitive to disturbance and were Phyllostominae. Aerial insectivores, belonging to Molossidae and Verspertilionidae, exhibited contrasting responses with *R*. *velilla* the only species showing sensitivity to disturbance^17^. This suggests that sensitivity to habitat disturbance was more a function of taxonomic group than guild. Although our data did not allow us to rigorously test the extent to which functional traits and phylogenetic constraints determine bats’ response to disturbance, they point to interesting patterns that warrant further attention.

Compared to incidence-based beta diversity, abundance-based beta diversity patterns were much more similar between the entire understory community and Phyllostomidae. For both groups, ß_Baselga B-C_ was highest between natural and degraded forest as opposed to natural and semi-natural forest as in the case of ß_sor_. These high scores can largely be explained by declines in the two dominant species, *A. fraterculus* and *D. rotundus*, which decreased by 58% and 79% between natural and degraded forest compared to 29% and 32% between natural and semi-natural forest, respectively. For both the entire community and Phyllostomidae, loss of individuals was the main process underlying abundance shifts between all disturbance levels, except semi-natural versus degraded forest in riparian habitat. Only in this transition were compositional differences driven less by overall loss and more by balanced variation in abundance, wherein individuals of some species were substituted by the same number of individuals of different species between disturbance levels^47^. Apart from this exception, the general pattern of declining abundance suggests a detrimental effect of land use on bat populations and hence a greater likelihood of local extinction for many species in degraded SDTF landscapes^19^. However, differences in relative abundance shifts between the whole understory community and Phyllostomidae among disturbance levels were obfuscated by the fact that the two dominant species were phyllostomids. If these two species along with the Phyllostominae are removed from consideration, it becomes apparent that most of the remaining species (i.e., *M*. *molossus*, *N*. *leporinus*, *G*. *soricina*, and *M. nigricans*) vary little in abundance across the disturbance gradient and thereby represent poor indicators of ecosystem integrity. In any case, conclusions regarding non-phyllostomid species in this study have to be made carefully given the low capture probability of some of these species with the use of understory mist nets.

Responses of *A. fraterculus* and *D. rotundus* underscored the utility of measures at the population and organismal level to evaluate ecosystem integrity. In support of the second hypothesis, both species decreased in abundance along the disturbance gradient, especially between semi-natural and degraded forest, although this difference was not significant for *D. rotundus* perhaps due to the refuge effect of riparian habitat^18^. Along with the disappearance of Phyllostominae species, the abrupt decline in *A. fraterculus* provided an indicator of the severity of disturbance in degraded sites. Previous studies report that *A. fraterculus* is one of the most common species along the Equatorial Pacific Coast, occurring in both primary and disturbed habitats^48–50^. According to Pinto et al.^50^, *A. fraterculus* tolerates disturbance given its capacity to use a variety of structures as roosting sites and ability to sustain itself on exotic fruits. In our study area, however, fruit plantations as well as most of the plants reportedly consumed by the species are scarce in degraded forest, which likely explains its low abundance. To our knowledge, this is the first study to show a significant decrease in *A. fraterculus* abundance with intensive land use.

In contrast to the second hypothesis, neither species’ body condition changed significantly with disturbance, although that of *D. rotundus* did deteriorate markedly from natural to semi-natural and degraded forests. Local ranchers consider the sanguivorous *D. rotundus* to be a threat to their livestock and elimination of its roosting sites is widespread. As a result, it is forced to find roosting sites in remote areas and expend energy foraging over large areas, which takes a toll on its body condition^51^. Decline in *A. fraterculus* abundance and *D. rotundus* body condition thus provide species-specific indicators of different levels of habitat disturbance in SDTF.

Community-based attributes along the disturbance gradient also varied by habitat type, underscoring the importance of comparing bat assemblages across different levels of habitat heterogeneity in human-modified SDTF landscapes. In support of the third hypothesis, three lines of evidence indicated that understory bats were more sensitive to disturbance in forest than riparian habitat, suggesting that the latter serves as a refuge for bat populations in degraded landscapes^27,28,52^. First, several species captured in riparian habitat were not detected in forest habitat in the semi-natural and degraded disturbance levels. Second, species loss was more important than species replacement in forest habitat relative to riparian habitat, especially for Phyllostomidae. Third, analogous to the previous result, abundance-based beta diversity was due primarily to balanced variation in species abundances in riparian habitat while loss of individuals was the driving process in forest habitat, especially between semi-natural and degraded forests. Riparian habitat offers a high diversity of chiropterophilic (i.e., pollination) and chiropterochoric (i.e., seed dispersal) resources to bats and provides roosting sites, water and corridors that facilitate their movement across the landscape^18,22,27^. In our study site, plant species characteristic of riparian zones such as *Ziziphus thyrsiflora*, *Trema micrantha, Celtis* spp., and *Ficus* spp. maintain at least a portion of their leaves during the dry season (Cueva E. pers. comm.), which is important for 11 of the 13 species in the study that use hollow trees or foliage as roosting sites^40,50,53,54^. However, capture probability may have increased in riparian corridors simply because individuals were forced to congregate there to find food or avoid predators, but in reality represented declining populations awaiting payment of an extinction debt^55^. Further research is needed to better understand bat population dynamics over larger temporal and spatial scales to determine whether riparian habitat supports viable populations in human-modified SDTF landscapes.

## Conclusions

Our findings suggest that understory phyllostomid species are a better indicator of habitat disturbance in SDTF than the understory bat community as a whole. Phyllostomidae richness, species composition, and species-specific measures were consistent and complementary indicators of habitat disturbance, whereas the understory bat community as a whole showed less sensitivity. The discrepancy between our findings and studies showing divergent disturbance responses within Phyllostomidae suggests a differential response of the family between tropical humid and tropical dry forests. In humid forests, high diversity of feeding guilds and large population sizes may blur a clear, uni-directional signal from the family^56^. In SDTFs, limited diversity and the rarity of many species may make Phyllostomidae highly susceptible to anthropic pressures, providing a clearer, more consistent signal of disturbance^18^. Phyllostominae, in particular, were shown to be sensitive to even moderate pressure, making this subfamily an exceptional bioindicator of ecosystem integrity in SDTF. However, the rareness of Phyllostominae as well as non-phyllostomid bats and the problems associated with low sample size emphasize the need for studies that combine complementary sampling methods (e.g., mist netting at different heights and acoustic monitoring) to detect bats and better understand the responses of these groups to SDTF disturbances^57,58^.

Phyllostomids’ response did vary somewhat depending on the disturbance level, habitat type, and attribute assessed, demonstrating the value of a multifaceted approach. In the transition from natural to semi-natural forest, the clearest indicators were the decline in richness and high beta diversity of both the entire understory community and Phyllostomidae, as well as loss of Phyllostominae species and deteriorating body condition of *D*. *rotundus*. Between semi-natural and degraded forest, the best indicators were an overall decline in phyllostomid richness and loss of phyllostomid species in forest habitat as opposed to riparian habitat. In addition, populations of *A. fraterculus* and *D. rotundus* suffered substantial declines. Between natural and degraded forest, the best indicators were the complete loss of Phyllostominae species and general declines in species abundances, as evidenced by large gradient changes in abundance-based beta diversity. Together, these results show that no single attribute encapsulated bats’ response to disturbance along the entire land-use gradient in SDTF. Rather, an approach that compared different community attributes, species-specific measures, taxonomic groups, and levels of habitat heterogeneity provided a more complete picture of their response to human impacts. Such a multipronged approach should be incorporated into monitoring strategies to enable comprehensive evaluation of the conservation status of bat communities and design strategies to help mitigate anthropogenic impacts in SDTFs.

## Methods

### Ethical approval

Bat captures were conducted in accordance with relevant guidelines and regulations as stipulated by the Environmental Ministry of Ecuador (research permit no. MAE-DNB-CM-2015–0016), which approved all sampling protocols. Captured bats were promptly removed from mist nets, identified, measured and released to minimize stress to individuals. No experimentation was conducted on bats. All capture events and animal handling were performed in accordance with the guidelines of the American Society of Mammalogists^59^.

### Study area and sampling sites

**Figure 6:**
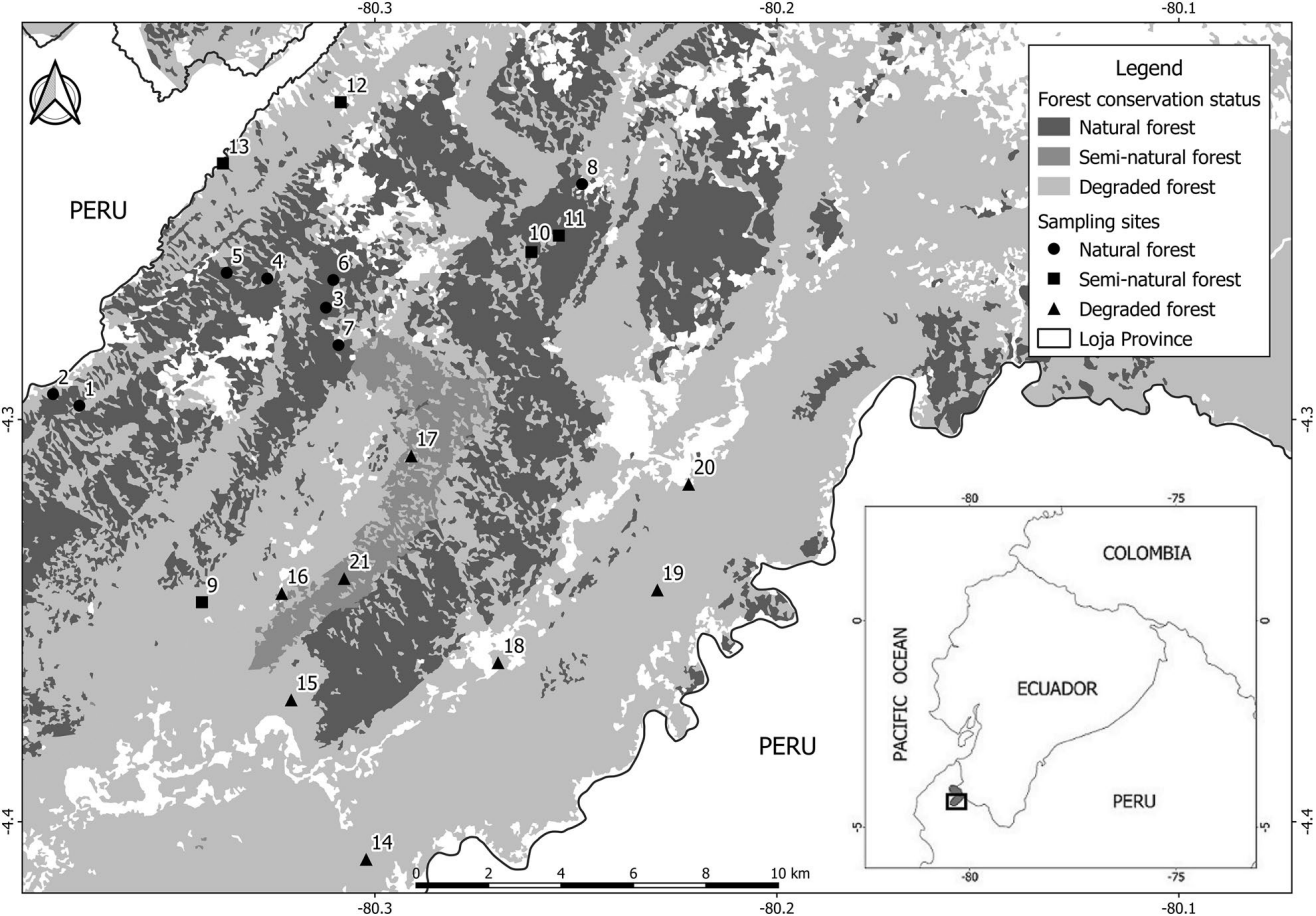
Study area and location of sampling sites. Numbers correspond to sites listed in Supplementary Table S1. The underlying map was constructed using QGIS version 2.14.20 (https://qgis.org/es/site/).

This study was conducted in Zapotillo, Loja Province, in southwestern Ecuador. The landscape consists of 120,797 hectares of SDTF and contains some of the largest and best-preserved remnants of this forest type in the entire biogeographical region of Pacific Coastal Ecuador^60^. Mean annual temperature varies from 18 to 26 °C and annual precipitation fluctuates between ca. 660 and 1300 mm^61^. The dry season lasts eight months from May to December, during which monthly precipitation rarely exceeds 10 mm, and the rainy season lasts four months from January to April^61^. Since the mid-twentieth century, anthropogenic activities have escalated in the area, particularly selective logging, agriculture, and intensive grazing by goats within forest remnants^62,63^. These pressures have impacted the biodiversity, structure and extent of SDTF in the region^35^, reducing mature forest to approximately one quarter of its original area^64^ (Fig. 6).

To determine the effect of habitat disturbance on bats within this landscape, we selected 21 sampling sites each representing one of three levels of forest disturbance following Cueva Ortiz and Chalán^65^ (Supplementary Table S1). Based on vegetation density and reflectance levels, these authors characterized SDTFs in the region as dense, semi-dense and sparse forest, hereafter referred to as natural (low anthropogenic pressure), semi-natural (moderate anthropogenic pressure), and degraded forest (intense anthropogenic pressure), respectively. Given that communities generally practice the same agricultural and livestock activities throughout the area, these levels of habitat disturbance are more a consequence of the length of time forests have been subjected to disturbance rather than the type or intensity of the pressure exerted^35^.

Sampling sites were located at least one kilometer apart from each other to ensure independence (Fig. 6). To minimize the influence of other disturbance levels, each site was selected so that the surrounding area was as homogeneous as possible.

### Bat sampling

Bats were surveyed using ground-level mist nets at the beginning of both the rainy season and dry season between May 2013 and November 2017. Surveys were carried out over 17 nights within each disturbance level—natural, semi-natural and degraded forest—for a total of 51 nights. At each site, six 3 × 9-m ground-level mist nets were arranged in two parallel rows separated by at least 50 m, with three nets each in forest and riparian habitat. Nets were placed 30 m apart within a row, with the result that approximately 90 linear meters were sampled in each habitat type. Deployed simultaneously in forest and riparian habitats, mist nets were opened for 3 h after dusk each night and inspected every 30 min. To identify recaptured individuals within the same two or three night sampling session at a given site, the hair was trimmed on the back of every bat captured prior to release^15,66^. All species capture data are available as Open Data (CC BY 3.0) in the Knowledge Network for Biocomplexity (KNB) Data Repository^67^.

### Data analysis

We used individual-based rarefaction and richness estimation based on Chao1 to compare richness of all species and phyllostomid species at two levels of habitat heterogeneity: (1) between sites to assess the effect of habitat disturbance represented by natural, semi-natural and degraded forest; and (2) within sites to compare forest and riparian habitats. To assess the completeness of each sample, we calculated the number of observed species as a portion of the total richness determined by the Chao1 estimator^45^. The use of this nonparametric estimator is recommended because it provides robust estimates of minimum richness for communities with many rare species by extrapolating the number of missing species in the observed data^43^. Significant differences in richness were identified by non-overlapping 95% confidence intervals of the rarefaction curves in cases where completeness was greater than 0.80. Diagrams of rank-abundance were used to visualize differences in bat community structure between forest and riparian habitats.

We evaluated beta diversity between disturbance levels in each habitat type for all species and phyllostomid species. Given the low number of captures at each site, we quantified pairwise dissimilarities between disturbance levels using the Sørenson (ß_sor_) and Simpson (ß_sim_) indices based on presence-absence, which provide independent measures of spatial species turnover and nestedness^46^, and the Baselga Bray–Curtis index based on abundance data (ß_Baselga B-C_), which provides independent measures of gradient and balanced changes in species abundances^47,68^. We also compared the body condition and abundance of the two most frequently captured species to evaluate their response to disturbance at the individual and population level. Body condition of each species was measured as the standardized residuals of a linear regression analyzing body mass as a function of forearm length^27^. The standardized residuals of this model are considered an appropriate index to assess body condition because they represent the non-allometric component of body size and thus enable the effect of condition to be distinguished from the effect of body size^69,70^. We performed a linear model (LM) to evaluate the effect of habitat disturbance on body condition and a generalized linear model (GLM) to analyze the effect of disturbance on abundance. Differences between disturbance levels were evaluated using a multi-test for abundance data, in which the reference level of the GLM was changed and p-values were multiplied by two to control the Type 1 error rate. We excluded young and pregnant individuals to avoid biases related to ontogeny and the additional weight of pregnant females^27^.

All analyses were performed in the R environment using the vegan package v.2.5–6^71^ for the rarefaction and beta diversity analysis and the base package^72^ for LM and GLM.

## Supplementary Information

Supplementary Information

## Data Availability

The dataset generated and analyzed in this study is available in the Knowledge Network for Biocomplexity (KNB) Data Repository with the identifier https://doi.org/10.5063/F1765CQJ.
