# Supplementary material for: A multifaceted approach to understanding bat community response to disturbance in a seasonally dry tropical forest
Source: Sci Rep. 2021 Mar 11;11:5667. doi: 10.1038/s41598-021-85066-z (PMC7970956; doi:10.1038/s41598-021-85066-z)
Supplement: Supplementary file 1 — Supplementary Information [file 41598_2021_85066_MOESM1_ESM.pdf]

Supplementary Material

A multifaceted approach to understanding bat community response to disturbance in a seasonally dry tropical forest

Darwin Valle, Daniel M. Griffith, Andrea Jara-Guerrero, Diego Armijos-Ojeda and Carlos I. Espinosa

*Corresponding author: *Daniel M. Griffith;* ***E-mail:*** dgriffith@utpl.edu.ec

This supplementary material contains:

Table S1. Sampling sites comprising a gradient of habitat disturbance in a human-modified seasonally dry tropical forest landscape of southwestern Ecuador.

Table S1. Sampling sites comprising a gradient of habitat disturbance in a human-modified seasonally dry tropical forest landscape of southwestern Ecuador.

Nº	Sampling sites	Level of habitat disturbance	Latitude	Longitude	Altitude (m)
1	Catanas 1	Natural forest	-4.29656	-80.37353	345
2	Catanas 2	Natural forest	-4.29369	-80.38008	338
3	Cabeza de Toro 1	Natural forest	-4.27217	-80.31218	410
4	Cabeza de Toro 2	Natural forest	-4.26492	-80.32679	419
5	Cabeza de Toro 3	Natural forest	-4.26350	-80.33687	480
6	Cabeza de Toro 4	Natural forest	-4.26528	-80.31037	433
7	Cabeza de Toro 5	Natural forest	-4.28149	-80.30907	478
8	Balsa Real 2	Natural forest	-4.24144	-80.24848	305
9	Totumitos	Semi-natural forest	-4.34537	-80.34303	212
10	Chilco	Semi-natural forest	-4.25835	-80.26107	270
11	Balsa Real 1	Semi-natural forest	-4.25425	-80.25425	367
12	La Manga	Semi-natural forest	-4.22106	-80.30852	519
13	El Salto	Semi-natural forest	-4.23629	-80.33781	401
14	Valle Hermoso	Degraded forest	-4.40934	-80.30218	180
15	Zapayal	Degraded forest	-4.36968	-80.32083	177
16	Chaquiro	Degraded forest	-4.34318	-80.32318	210
17	Malvas 1	Degraded forest	-4.30906	-80.29096	310
18	El Carrizo	Degraded forest	-4.36041	-80.26938	194
19	Zapotillo	Degraded forest	-4.34233	-80.22975	282
20	Ceiba Chica	Degraded forest	-4.31602	-80.22196	242
21	Malvas 2	Degraded forest	-4.33948	-80.30765	251
